# Automated Intraoperative Short Messaging Service Updates: Quality Improvement Initiative to Relieve Caregivers’ Worries

**DOI:** 10.2196/36208

**Published:** 2022-05-06

**Authors:** Alexandre Mignault, Éric Tchouaket Nguemeleu, Stephanie Robins, Éric Maillet, Edwige Matetsa, Stéphane Dupuis

**Affiliations:** 1 Bloc Opératoire Centre hospitalier de l’Université de Montréal Montréal, QC Canada; 2 Département des sciences infirmières Université du Québec en Outaouais St-Jérome, QC Canada; 3 École des sciences infirmières Faculté de médecine et des sciences de la santé Université de Sherbrooke Sherbrooke, QC Canada

**Keywords:** COVID-19, surgery, intraoperative, OR nurse, communication, technology, short messaging service, SMS, text message, caregiver, anxiety, perioperative, surgical, surgical procedure, mHealth, mental health, digital health, digital health care

## Abstract

**Background:**

Undergoing a surgical procedure is anxiety provoking for patients and their caregivers. During the intraoperative period, caregivers seek out informational updates from health care professionals, a situation complicated by COVID-19 health measures that require caregivers to wait outside the hospital. Short messaging service (SMS)-based communication that allows caregivers to follow their loved ones through surgery has shown promise in relieving anxiety and improving satisfaction with overall care. This form of communication is also well accepted by health care professionals and may be effective at relieving staff burden.

**Objective:**

Here, we describe a quality improvement initiative of a standardized and integrated intraoperative SMS-based system to improve communication between surgical teams and caregivers. The main goal was to improve satisfaction with care, while the secondary goal was to reduce caregiver anxiety.

**Methods:**

The initiative followed the framework of the Model for Improvement. A large tertiary care hospital offered the SMS to caregivers who were waiting for loved ones undergoing surgery. SMS messages were integrated into the clinical information system software and sent at key points during the surgical journey to phone numbers provided by caregivers. A satisfaction survey was sent to caregivers 1 business day after surgery. Data were collected between February 16 and July 14, 2021.

**Results:**

Of the 8129 surgeries scheduled, caregivers waiting for 6149 (75.6%) surgeries agreed to receive SMS messages. A total of 34,129 messages were sent. The satisfaction survey was completed by 2088 (34%) of the 6149 caregivers. Satisfaction with messages was high, with the majority of respondents reporting that the messages received were adequate (1476/2085, 70.8%), clear (1545/2077, 74.4%), informative (1488/2078, 71.6%), and met their needs (1234/2077, 59.4%). The overall satisfaction score was high (4.5 out of 5), and caregivers reported that receiving text messages resulted in a reduction in anxiety (score=8.2 out of 10). Technical errors were reported by 69 (3.3%) caregivers. Suggestions for improvements included having messages sent more often; providing greater patient details, including the patient’s health status; and the service being offered in other languages.

**Conclusions:**

This digital health initiative provided SMS messages that were systematically sent to caregivers waiting for their loved ones undergoing surgery, just as COVID-19 restrictions began preventing waiting onsite. The messages were used across 15 surgical specialties and have since been implemented hospital-wide. Digital health care innovations have the capacity to improve family-centered communication; what patients and their families find useful and appreciate will ultimately determine their success.

## Introduction

### Background

Surgery, whether elective or emergent, is a distressing medical procedure that evokes high levels of anxiety in both patients and caregivers [1-3]. More than 1 million surgical interventions were performed in Canada in 2020 [4]; these procedures implicate family members and caregivers as requisite accompaniers. Separated from their loved ones during the intraoperative period, caregivers experience distress, helplessness, fear, loneliness, frustration, and uncertainty, as well as physiological responses, such as increased heart rate, impaired sleep, and restlessness [5-7]. Although caregivers previously waited in the surgical waiting area, with the arrival of the COVID-19 pandemic and mandatory hygiene measures, most are now required to wait off-site with only remote access to surgical staff for updates [8].

Family members anxiously seek informational updates about their loved one’s status [7,9], but the intraoperative time frame is often the most difficult moment to provide such details. Progress reports are effective at relieving the distress felt by caregivers during surgery and contribute to overall satisfaction with care [10,11]. In fact, surgeons consider the main purpose of their intraoperative communication with family members to be the reduction in anxiety [12]. During these moments, surgeons report that the surgical details are not remembered by family members and caregivers, whose primary concern is to know whether their loved one is alive and awake.

The importance of including caregivers in the surgical conversation reflects *information sharing*, 1 of the core concepts of patient- and family-centered care (PFCC) [13]. PFCC has been shown to lead to improved patient health outcomes, a better overall experience of care, and a wiser allocation of resources [13]. Fostering effective intraoperative communication to fulfill PFCC has become a priority in the surgical setting, where surgeons, anesthetists, nurses, and receptionists are often solicited for information. Surgical nurse liaisons are described in the scientific literature as being the link between family and the operating room (OR) and are often responsible for providing specific, ongoing, and predictable information on the day of surgery [14-18].

Hospitals have supplemented face-to-face perioperative consultations with other modes of information provision. These include using volunteers for support with navigating the hospital, providing informational cards [19], installing electronic patient status boards in waiting rooms [20], showing videos that describe the surgery [21,22], and allowing a 5-minute postanesthesia care unit (PACU) visit between caregivers and patients [23]. Although speaking with a member of the surgical team remains the gold standard, a 2016 study by Heath et al [18] found that families receiving intraoperative updates from a nurse were equally satisfied if they received them in person or by telephone. Indeed, the authors suggested that telephone calls provided more individualized care and privacy for family members. As a result, they could wait and receive news wherever they preferred.

Mobile health (mHealth) solutions in the field of surgery have grown as the use of mobile phones has become nearly universal [24,25]. Recent reviews examine mobile app–based and short messaging service (SMS)-based interventions in the management of surgical patients [26-28]. The overall findings reveal that SMS-based perioperative communication is acceptable, efficient, and effective for patients, caregivers, and health care providers. Furthermore, the interventions demonstrate positive results, including reduced anxiety, increased adherence to treatment, improved symptom monitoring, better pain management, increased satisfaction with care, and lower postoperative readmission rates [26,27]. Importantly, they also provide continuity of care in the preoperative-to-postoperative window [11,29-31].

Studies that use SMS-based communication to update family members and other caregivers during the intraoperative period are limited yet offer compelling evidence of their value as they produce positive outcomes [32-36]. Gordon et al [32] carried out a multicenter prospective study that connected surgical patients to any number of individuals designated as a contact. This person received 7 emails or SMS updates. Two days postoperatively, patients, message recipients, and surgical staff completed a satisfaction survey. A large majority of patients (74%) endorsed the program as being an “improved hospital experience,” while 96% of the message recipients claimed they “felt more connected to their loved ones during surgery.” For the surgical team, 87% found it to be “useful and efficient.” Wieck et al [35] describe the integration of pager-based SMS updates in a children’s hospital as part of an effort to streamline communication with families. Families were paged with 4 updates during surgery. Satisfaction with information increased over 30% for families, and 96% of nurses felt that “patients' families were getting the information that they desired.” In contrast, Howe et al [36] tested the effect of pager-based updates using a randomized controlled trial of adults admitted for orthopedic surgery. The families in the control group received care as usual, while the intervention group received a text message at the beginning, middle, and end of surgery. The intervention group experienced lower levels of anxiety and higher levels of satisfaction with the information provided compared to the control group. In 2016, Kwan et al [33], in a nonrandomized prospective survey, measured the perioperative level of anxiety in parents whose children were undergoing spinal surgery. In the intervention group, parents received 10 SMS updates every 10-20 minutes during surgery, while the control group received treatment as usual. The intervention group had significantly lower measures of anxiety both during surgery and postoperatively. Similarly, Poudel et al [34], using a randomized single-blinded prospective study, measured anxiety in family members who were waiting for loved ones undergoing oncologic surgery. The control group received care as usual, while the intervention group was provided with intraoperative SMS updates at 5 times points during surgery. The SMS group had significantly lower anxiety scores at 1 hour into surgery and at surgery completion compared to the control group.

Patients, health care providers, and message recipients find SMS-based updates of surgical milestones to be acceptable, useful, and anxiolytic. In the context of the COVID-19 pandemic, mobile apps that allow caregivers to follow their loved ones through surgery are beginning to be offered commercially and are being integrated into medical centers [37]. However, commercially available apps raise concerns surrounding privacy, security, and reliability. Furthermore, context-specific mobile apps are not suited to provide a standardized system of messaging that translates into sustainable interventions.

### Objectives

This paper describes a quality improvement initiative that consisted of the implementation of a standardized and sustainable intraoperative SMS-based system that improves communication between surgical teams and caregivers [38]. Specifically, this initiative aims to improve caregiver satisfaction with care and reduce caregiver anxiety during the intraoperative period.

## Methods

### Clinical Setting

This quality improvement initiative was undertaken at the Centre hospitalier de l’Université de Montréal (CHUM) in Montreal, Quebec, Canada. This newly constructed hospital represents the modernization and centralization of 3 separate hospitals where, between 2015 and 2020, an average of 24,000 surgeries occurred each year. The hospital runs a total of 36 surgery rooms spread over 2 floors, and 16 surgical specialties are practiced in the new center. For this project, most specialties were involved; only ophthalmology, obstetrics, the burn center, and emergent surgeries were not included in this initial phase. Approval for the initiative was obtained from the director of professional services and the associate director of academic and university affairs of the hospital. This quality improvement initiative followed the framework set out by the Model for Improvement originally described by the Associates in Process Improvement [39,40]. The process involves forming a team; setting an aim; selecting measures and changing them as required or suggested; pilot-testing the initiative; implementing changes; and spreading the change more globally. CHUM supports this cycle of innovation for creating value in health care (eg, improving patient care as well as staff and team experiences, optimizing resources, collaborating with educators) [41].

### Ethical Considerations

This quality improvement initiative did not require CHUM Institutional Research Board review. All caregivers who participated in this study were treated in accordance with the Declaration of Helsinki (7th revision 2013). Participants provided verbal consent that their caregiver receive SMS messages from the digital platform and were able to opt out at any time without affecting the standard of care. Participant (caregiver) information was not associated with the data collected for the purpose of this initiative, and personal patient information was not collected, transferred, or published. These measures were put in place to maintain the right to privacy and confidentiality.

### Procedures

A member of the surgical team described the SMS system to caregivers and how they could receive intraoperative messages if they so desired. This was done during surgery scheduling or at admission for surgery. Messages were provided as a parallel system to standard care and were not included in the medical health record of the patient. Caregivers provided a phone number to the staff and were told that unidirectional updates would be sent during specific points during surgery and that the last update would indicate the unit where their loved one was recovering or when they would be discharged. Caregivers were required to wait off the hospital premises during surgery due to COVID-19 restrictions. A final message was sent to the caregivers 1 business day after surgery to invite them to complete a survey using the online platform Lime Survey. No reminders were sent.

The system (including messages and the satisfaction survey) was pilot-tested for reliability and acceptability between January 11 and February 14, 2021. The research team and 2 staff members reviewed the patient intake process, the functionality of the digital platform, and the content of the caregivers’ responses. From a total of 884 participating caregivers, 404 (45.7%) completed the questionnaire. Refinement of the initiative occurred at this stage. One SMS message was removed from the surgical updates as it was deemed unnecessary. As caregivers noted (in open-ended questions of the satisfaction questionnaire) that the SMS messages reduced their anxiety, a single question on anxiety was added, as has been done by others [42,43]. Finally, 1 question that provided an open-ended choice for improvements on the SMS messages was made into a drop-down menu for commonly noted suggestions from this pilot phase, with 1 additional open comment box. Data collection of survey responses took place between February 16 and July 14, 2021.

### Development of the SMS System and Messages

SMS messages and their send times were integrated into the clinical information system software Centricity Opera (General Electric Healthcare) [44]. These modifications to Centricity Opera were made by working in close collaboration with the company that provides the software. Messages were sent from Centricity Opera to the Application Programming Interface company Twilio [45], which then transmitted the messages to caregivers.

The wording of all SMS messages was developed by the surgical staff working group (the research team). For the initial phase, messages were only offered in French. The text was then reviewed in collaboration with the hospital’s communication department and edited to consider privacy and to ensure messages were written in clear, concise, and accessible language. SMS messages provided resources, including a link to the hospital’s appointment center and a telephone help line with a 24-hour-a-day nurse available to discuss patient concerns. Wording for 2 additional SMS messages was prepared: (1) in the event caregivers needed to come to the hospital during the time of COVID-19-mandated curfew hours, the message provided the necessary medical authorization to travel during curfew, and (2) in the case of a power failure, a customizable message was created such that it could be sent once the system functioning returned, noting that messages may have been interrupted. All SMS messages are presented in Table 1.

**Figure 1:**
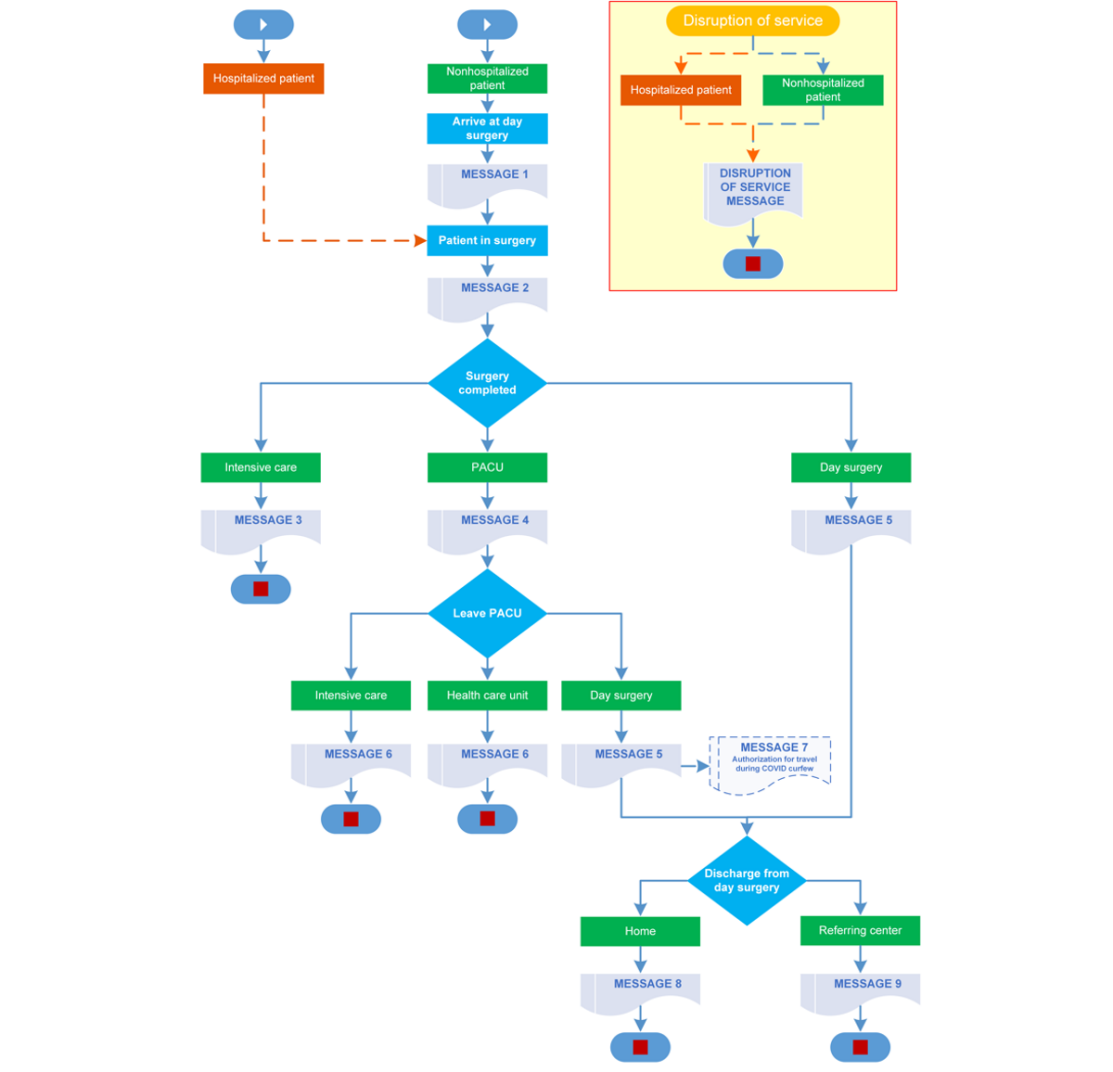
SMS Messages sent during patient's surgical trajectory.

The timing of SMS message delivery was decided upon by the research team. Updates were sent out as patients traveled through checkpoints considered key times in the surgical trajectory (see Figure 1). Although the messages were labeled for internal identification using numbers, these were not seen by caregivers. In fact, each surgical journey differed by patient, depending on their condition. For example, a patient may have come to surgery from within the hospital, been operated upon, gone through the PACU, and then been sent to the care unit. The appropriate and relevant messages received by their caregivers would be identified internally as message 2, followed by message 4 and then message 6.

**Table 1:** SMS^a^ messages sent to caregivers during key times during surgery.

Message	Content
Message 1	CHUM^b^ Day Surgery One of your loved ones wishes to keep you informed of their progress during their day of surgery. The patient has just arrived at the day surgery department. This is the first in a series of messages intended to keep you informed of the progress of his or her surgery. Please note that for reasons of confidentiality we do not transmit any medical information via the text messaging system. CHUM day surgery +1 (514) XXX-XXXX https://repertoire.chumontreal.qc.ca/fiches/chirurgie-dun-jour
Message 2	CHUM OR^c^ Your loved one is presently in the surgery room. Surgery will begin shortly. You will receive an SMS once the surgery is complete.
Message 3	CHUM PACU^d^ Your loved one’s surgical procedure is complete. He or she is now on their way to the care unit. This is the last message you will receive from the OR team.
Message 4	CHUM PACU The surgery is complete. Your loved one is currently in the PACU. You will receive the next SMS when he/she has completed the post-surgery safety monitoring period. Please note that since the PACU is a sterile area, visits are not permitted.
Message 5	CHUM Day Surgery Your loved one has returned to the day surgery unit. You will receive a message when he or she has met the discharge criteria. COVID-19 restrictions: you must wait for the nurse's call before coming to pick up your loved one.
Message 6	CHUM PACU The surgical procedure of your loved one is complete, and he or she is now on their way to the care unit. This is the last message you will receive from the OR team.
Message 7	For travel during COVID curfew: CHUM authorization After receiving the call from the nurse, use the attached authorisation to justify your trip to the CHUM.
Authorization for discharge	Accompanying a patient admitted at the CHUM during the curfew decreed by the Quebec government. You will find below an authorization from the Centre hospitalier de l'Université de Montréal authorizing you to travel during curfew hours for the sole purpose of picking up your loved one at the hospital when he or she is ready to go home. Be sure to keep this message until you return home. To whom it may concern, this message certifies that the bearer is the escort authorized by a CHUM patient who was discharged from the hospital following surgery today. To verify the authenticity of this discharge certificate, contact the hospital department at +1 514-XXX-XXXX. CHUM, 1051 Sanguinet Street, Montreal, QC H2X 3E4.
Message 8	CHUM Day Surgery Your loved one has completed his or her surgical journey and has met the criteria for discharge. He or she can now leave the hospital. Report to the Departure Lounge (Pavilion C - Ground Floor) or to the pickup area as directed by the nurse. This is the last message you will receive from the OR team. Health file: https://www.chumontreal.qc.ca/en/fiche/who-can-i-ask-if-i-have-questions-about-my-health Are you worried or do you need advice following your visit to the CHUM? Dial: +1 (514) XXX-XXXX.
Message 9	CHUM Day Surgery Your loved one’s surgical procedure is complete, and he or she has met all discharge criteria. He or she is now being transferred to the referring center. This is the last message you will receive from the OR team. Health Sheet: https://www.chumontreal.qc.ca/en/fiche/who-can-i-ask-if-i-have-questions-about-my-health Are you worried or do you need advice following your visit to the CHUM? Dial: +1 (514) XXX-XXXX. To reach the appointment centre at the CHUM: +1 (514) XXX-XXXX or +1 (855)-XXX-XXXX.
Notice of disruption	Notice of Disruption of CHUM Text Messaging Service Due to a disruption in our text messaging system, you may have experienced difficulties in receiving messages from the CHUM concerning your loved one. We apologize for the inconvenience. The messaging service has now been restored. Thank you for your understanding. The CHUM OR team
CHUM survey	Hello Our files indicate that you received SMS updates of your loved one during their surgical journey. We are sending you a survey regarding your satisfaction with the different SMS you received. The survey is confidential. Thank you for your time. CHUM Team - Client satisfaction team

^a^SMS: short messaging service.

^b^CHUM: Centre hospitalier de l’Université de Montréal.

^c^OR: operating room.

^d^PACU: postanesthesia care unit.

### Questionnaire

A satisfaction survey was developed by our team consisting of 10 self-reported items, 9 (90%) of which were used in this analysis.

One question asked whether respondents noticed that the day surgery contact number was included in their first message, to which they were able to answer either yes or no. This question was included to see whether caregivers were able to absorb the information provided and make effective use of resources.

Four items measured satisfaction with the messages and asked whether (1) the number of SMS messages received was adequate, (2) the messages delivered were clear, (3) the messages delivered kept respondents informed about the progress of their loved ones' operation, and (4) the information provided in the messages during the day met the respondent’s needs and expectations. Response choices consisted of a 4-point forced Likert scale: 1=completely agree, 2=agree, 3=disagree, and 4=completely disagree.

To verify the adequacy of the information included in SMS updates, 1 item queried whether respondents needed to contact the day surgery service despite having received SMS messages; response items were either yes or no. Those who responded yes were offered a menu of reasons why they contacted the service, including “To find out a room number,” “For information about the length of the operation,” “For additional information about the operation,” “For information about the condition of my loved one’s health,” “For information concerning discharge time,” “For the address of the hospital,” and 1 open field to describe “Other.”

In line with Howe et al [36], overall satisfaction was assessed with the question “On a scale of 1 to 5, how would you rate your overall satisfaction with the SMS application? (with 1 being completely dissatisfied and 5 being completely satisfied)?” One open-ended question asked respondents whether they had any suggestions or comments following their experience with the messaging system.

Anxiety was measured by the single question “On a scale of 1 to 10, to what level did receiving text messages reduce your anxiety about your loved one's surgical journey? (with 1 being not at all reduced and 10 being greatly reduced)?”

### Data Analysis

Anonymized data were exported from Lime Survey into Microsoft Excel for analysis. Results are expressed using descriptive statistics, frequencies, percentages, and mean scores. Spearman correlation was used to measure the association between total satisfaction and reduction in anxiety. “Other” reasons for having to contact the day surgery were described. Responses to the open-ended question that asked for comments or suggestions about the platform were analyzed by the team; similar items were coded and grouped into unique categories; frequencies are reported for these categories. Missing data were approximately 1%, and thus case-wise deletion was used to obtain all descriptive statistics [46,47].

## Results

### Users and Communication

Of the 8129 surgeries scheduled between January 14 and July 13, 2021, caregivers waiting for 6149 (75.6% participation rate) surgeries agreed to use the SMS system. A total of 34,129 messages were sent, resulting in an average of 5.6 messages per user. From 2088 respondents, 69 (3.3%) errors were considered technical issues (ie, software malfunction). Negative feedback included messages sent at a time that did not correspond with the surgical schedule, were missing, or were repeated. The staff may have incorrectly entered the time of surgery in the software; at other times, the origin of the error was not known. A few caregivers reported they did not receive SMS messages, an error that was determined to be due to incorrect phone numbers being linked to the caregivers, due to either caregiver or staff error in providing or recording the phone numbers. Information technology network downtime and power outages occurred twice; caregivers received the message drafted for this purpose, although some respondents noted the delay and a lack of communication in the open-ended question on satisfaction with the service. Other errors were determined to be a lack of human care coordination with the SMS messages. Caregivers reported mistimed instructions for pick-up of patients (too early) or an absence of expected communication from the nurse postoperatively.

### Level of Caregiver Satisfaction and Anxiety

The satisfaction questionnaire was sent to all 6149 respondents 1 working day after surgery, of which 2088 (34%) completed it. A majority of respondents (1511/2054, 73.6%) endorsed yes (they had seen the phone number provided in the first SMS sent) versus no (543/2054, 26.4%).

Satisfaction with messages was high, with the majority of respondents claiming they completely agreed that the number of messages received was adequate (1476/2085, 70.8%), clear (1545/2077, 74.4%), informative (1488/2078, 71.6%), and met their needs (1234/2077, 59.4%); see Table 2.

Approximately 1 (20%) in 5 caregivers (425/2055, 20.7%) needed to contact the day surgery unit. Reasons for this communication are described in Table 3.

Other reasons (78/425, 18.4%) for contacting the OR included questions or comments pertaining to surgery cancellations or delays, longer-than-normal perceived length between SMS messages, permission to visit the patient, planning of travel for patient transport home, clarification of messages or the SMS process, worry and stress about the patient, and trouble with the SMS system. Overall satisfaction with the app had an average score of 4.5 out of 5 (2041/2088, 97.7%).

In response to how SMS messages reduced anxiety in relation to the patient’s surgical journey, caregivers reported an average score of 8.2 out of 10 (2046/2088, 98%), where 10 represented “greatly reduced.” Spearman correlation revealed that the overall score in satisfaction was highly correlated with the reduction in anxiety (r_s_=0.608, *P*<.001).

**Table 2:** Caregiver satisfaction with SMS^a^ messages.

Item	Completely agree, n (%)	Agree, n (%)	Disagree, n (%)	Completely disagree, n (%)	Total, N
The number of SMS messages received was adequate.	1476 (70.8)	501 (24.0)	73 (3.5)	35 (1.7)	2085
The messages delivered were clear.	1545 (74.4)	468 (22.5)	45 (2.2)	19 (0.9)	2077
The messages delivered kept caregivers informed about the progress of their loved one’s operation.	1488 (71.6)	475 (22.9)	89 (4.3)	26 (1.2)	2078
The information provided in the messages during the day met the caregiver’s expectations and needs.	1234 (59.4)	651 (31.3)	147 (7.1)	45 (2.2)	2077

^a^SMS: short messaging service.

**Table 3:** Reasons caregivers contacted day surgery despite having received SMS^a^ messages (N=425).

Reason	Caregivers, n (%)^b^
To find out a room number	56 (13.2)
For information about the length of the surgery	89 (20.9)
For additional information about the surgery	126 (29.6)
For information about the condition of my loved one’s health	217 (51.1)
For information concerning discharge time	113 (26.6)
For the address of the hospital	7 (1.6)
Other	78 (18.4)

^a^SMS: short messaging service.

^b^Categories are not mutually exclusive, and thus percentages do not add up to 100%.

### Caregiver Comments

The majority of respondents (1360/2088, 65.1%) answered the open-ended question regarding their experience; comments were subsequently collapsed into 7 unique categories and 20 subcategories to obtain a total of 2078 comments (see Table 4). Caregivers provided feedback not only for the SMS service but also for their experience worldwide.

**Table 4:** Subcategories of commentary provided by caregivers (N=2078).

Category		Caregivers, n (%)
**Positive feedback (n=1293, 62.2%)**		
	Positive comments, thanks, and congratulations	492 (38.0)
	Specific positive feedback on the SMS^a^ system	633 (49.0)
	Positive feedback on the surgical experience	87 (6.7)
	Reduced anxiety	81 (6.3)
**Desire for more information (n=352, 17.0%)**		
	Would have liked to know the room number of their loved one	43 (12.2)
	Would like to know state of health of the patient	152 (43.2)
	Surgeon’s call important	15 (4.3)
	Would like to know how long each segment of wait is	93 (26.4)
	Discharge information not detailed or precise enough	49 (13.9)
**Negative feedback (n=139, 6.7%)**		
	Was stressful	12 (8.7)
	Mistimed SMS	64 (46.0)
	Delays between SMS messages too long	48 (34.5)
	Dissatisfied with the message system	15 (10.8)
**Software error (n=69, 3.3%)**		
	Number or delivery of texts incorrect	69 (100.0)
**Constructive criticism (n=58, 2.8%)**		
	Messages need to be clarified.	27 (46.6)
	Messages feel impersonal in their tone.	21 (36.2)
	The SMS should also be provided in English.	10 (17.2)
**Dissatisfied with experience at the OR^b^ (n=38, 1.8%)**		
	Surgery delayed or cancelled	38 (100.0)
**Incomplete comments or other issues (n=129, 6.2%)**		
	Unclear or incomplete messages	55 (42.6)
	Comments about other issues or departments (eg, security)	74 (57.4)

^a^SMS: short messaging service.

^b^OR: operating room.

## Discussion

### Principal Findings

The purpose of this paper was to describe an SMS-based digital health initiative that aims to improve communication between surgical teams and caregivers during the time of surgery. The SMS platform was specifically designed to improve caregiver satisfaction with care and to reduce caregiver anxiety. Caregiver reports of satisfaction with the messages and the initiative were high. Caregivers also reported a positive effect on anxiety reduction and offered constructive feedback on how to improve the quality, content, and method of delivery of information.

The results of this study confirm that integrating a standardized system of intraoperative messages in the clinical information system of an OR can enable SMS updates that can be sent in real time to those waiting for loved ones undergoing surgery. Unlike other context-specific innovations, this initiative was integrated with the existing hospital’s software infrastructure and was thus generalizable to other settings using clinical information system management software. SMS communication for surgical updates is now a permanent service being offered throughout the CHUM.

This project and its outcomes support the vision of the World Health Organization (WHO) Global Strategy on Digital Health for 2020-2025 [48], which states that digital innovations will be valued and adopted if they are accessible and sustainable, increase efficiency in the delivery of care, and protect the privacy of patient health information. This initiative was piloted and then implemented for testing just as COVID-19 restrictions were rendering access to hospital wait areas impossible. Thousands of caregivers were able to receive SMS communication about their loved ones, in addition to care as usual. To the best of our knowledge, this study has the largest sample size to date for intraoperative messaging.

### Satisfaction With Messages

Overall, caregiver satisfaction was high. Due to the wide range of surgical specialties involved in this project, there were many combinations of patient trajectories and timing (see Figure 1), and thus, the number and frequency of messages differed. Despite this, over 90% of respondents agreed or completely agreed that the information provided was clear and adequate, kept them informed about their loved one’s progress, and met their expectations. Of the caregivers who needed to contact the day surgery, many had concerns with regard to the evolution of surgery and the condition of their loved one’s health (see Table 3). The SMS messages did not contain individualized health information in order to avoid a breach of patient privacy.

Caregiver worries were addressed by talking with a staff member, providing insight into avenues for future modifications to the timing and content of updates. Updates may be better received if they can be provided more frequently and with more patient-specific content. This recommendation was specifically noted by caregivers in their suggestions for improvements. In line with this commentary are those suggestions made by caregivers participating in an intervention that provided intensive care unit patients’ families with daily updates by SMS [31]. In 32.3% of participants, feedback regarding the updates was that they contained “sparse and not very concrete” information about their loved ones [31]. Patient information that is curated was described by Globus et al [49]. Parents of infants in neonatal intensive care—who undergo extremely stressful separations from their babies daily—received SMS updates once a day that included information that was both nonmedical (eg, location of crib) and medical (eg, babies’ weight, procedures performed). As a result, parents reported feeling more at ease in approaching medical staff and more satisfied with regular information provision concerning their infant’s medical status. Thus, without dehumanizing the patient-provider experience, SMS messages have the power to contribute to the continuum of care and empower caregivers with information.

### Anxiety

Those who wait are heavily emotionally invested in the information they seek and report the wait as being a time of constant anxiety and exhausting vigilance, which is diminished slightly by human interaction and, then, the end of surgery [6,50]. Overall, caregiver anxiety was reduced to a large extent (score=8.2 out of 10) by receiving communication from the OR, suggesting that the messages were effective at reducing intraoperative stress. This was the second goal of the initiative. This effect was confirmed in the commentary that was freely provided in open-ended answers and was part of the reason a measure of anxiety was included after the pilot phase. The positive effect of reducing anxiety using SMS intraoperative updates has been demonstrated in a range of surgical specialties using controlled studies [33,34,36]; here, we confirmed their findings. It is important to address anxiety to reduce adverse outcomes seen in caregivers that persist postoperatively. These include fear of death of a loved one, frustration, anger, guilt, and other lasting psychological and physical disturbances [7,51-53].

### Global Satisfaction

The reduction in anxiety seen in this project may have been an essential driver in the overall satisfaction scores of 90% (or 4.5 out of 5), as the 2 were highly correlated. This level of satisfaction is in line with Gordon et al [32], where 94.3% of caregivers responded they “enjoyed this software” in response to receiving 7 email or SMS customized intraoperative updates. Receiving mobile-based messages also offers caregivers the freedom to better plan their wait and may thus influence their overall experience. Prior to COVID-19-mandated off-site waiting, leaving the hospital was reported as a coping strategy used by parents of oncology patients who could not bear to sit in a waiting room during their child’s surgery [7]. Instead, while waiting for news, they filled “unoccupied” time with “occupied” time [54] and fared better in terms of anxiety and distress, as reported by parents who stayed at the hospital.

### Future Directions

Evidence from future controlled and qualitative studies may result in intraoperative text-based communication systems such as this one becoming a permanent adjunct to the standard of care. Aside from clinical applications, the platform may also serve as a skeleton upon which other perioperative communication interventions can extend their research capacity. For example, Farias et al [29] tested a perioperative communication and support system that delivered messages by SMS to parents of children undergoing tonsillectomy. Parents were contacted both before and after, but not during, the surgery. Interviews with parents revealed that even though the messages were automated, parents felt continuously supported and that they would have appreciated receiving more information and more messages. Adding the intraoperative period using a system such as the one installed at the CHUM may have been well received by the parents.

### Limitations

This initiative had a few notable limitations. First, for caregivers who waited for surgeries that spanned many hours, receiving messages at strategic trajectory-related time points may not have satisfied the desire to know how the surgical procedure itself was evolving. Future iterations should include updates that are sent at a minimal interval in order to prevent delay-related anxiety and concern in those who are waiting. Second, caregivers were required to understand written French and own and be comfortable using a network-supported mobile phone capable of receiving text-based messages. However, a quarter of those approached opted not to use the SMS platform. It may have been that among those caregivers who declined, some were not able to communicate in French or at ease with digital technology. To attain *digital health equity* for patient health initiatives, designs should consider socioeconomic determinants of health [55]. For example, not all health care users have access to technology or the eHealth literacy needed to navigate digital tools. There were no demographic and socioeconomic data collected from caregivers that may have provided insight into how the technology was received and appreciated. Collecting this data in the future will allow for more nuanced analyses to identify predictors of acceptability, satisfaction, and anxiety reduction. Third, our response rate was 34% (n=2088), which is approximately 10% lower than average controlled studies with surgical patients and health care providers [56] and online surveys generally [57]. A higher response rate from a future study using the same platform will help inform the outcomes reported by this cohort [58]. Fourth, no control group was used, nor were health care providers or administrators included at this stage. Future assessments of this service would benefit from a control group as well as professionals to help assess degrees of effect and acceptability and to gather feedback for improvement. Future research planned by the team should include a 2-armed randomized prospective study to determine the impact of this innovation. Finally, errors in message delivery were reported by 69 (3.3%) of 2088 caregivers. Regulatory agencies require that hospitals maintain mechanisms to protect against accidental disclosure or loss of patient health information [59,60]. Thus, although the SMS messages did not reveal any personal information, careful staff training and system checks should be put in place to help eliminate instances of messages being sent at the wrong surgical time, to an incorrect number, or not at all.

### Conclusions

Due to the increasing prevalence of smartphone ownership, text-based messaging has become an indispensable tool in patient and caregiver surgical care [26]. Here, we described an innovative SMS-based communication system to keep caregivers, family members, and friends up to date on the surgical trajectory of their loved ones. This initiative has informed best practices for hospital-wide implementation and has provided evidence-based data for a scaled-up version of SMS communication in a surgical setting in any hospital. Feasible and acceptable, SMS messages are likely to be a vital adjunct to in-person communication, as they have the potential to reduce the burden of health care professionals and increase efficiency. Importantly, they can also satisfy the tenets of PFCC and contribute to improved overall health care. In the context of COVID-19, adapting to technologically supported methods of safely sharing patient information will be paramount.

## Appendix

Multimedia Appendix 1 Satisfaction survey: original French.

Multimedia Appendix 2 Satisfaction survey: English translation.

## Abbreviations

CHUM: Centre hospitalier de l’Université de Montréal
OR: operating room
PACU: postanesthesia care unit
PFCC: patient- and family-centered care
SMS: short messaging service

## References

[1] Davis Y, Perham M, Hurd AM, Jagersky R, Gorman WJ, Lynch-Carlson D, Senseney D (2014). Patient and family member needs during the perioperative period. J Perianesth Nurs.

[2] Stamenkovic DM, Rancic NK, Latas MB, Neskovic V, Rondovic GM, Wu JD, Cattano D (2018). Preoperative anxiety and implications on postoperative recovery: what can we do to change our history. Minerva Anestesiol.

[3] Hui WJ, Pikkarainen M, Nah SA, Nah SNJ, Pölkki T, Wang W, He H (2020). Parental experiences while waiting for children undergoing surgery in Singapore. J Pediatr Nurs.

[4] Information and Privacy Commissioner of Ontario (2009). The Surgical Safety Checklist: A Must for Hospitals Performing Surgery.

[5] Colussi G, Frutos E, Rapisarda R, Sommer J, Descalzo J, Plazzotta F, Luna D (2021). Information needs at the O.R. waiting room. Stud Health Technol Inform.

[6] Trimm DR, Sanford JT (2010). The process of family waiting during surgery. J Fam Nurs.

[7] Gabriel MG, Wakefield CE, Vetsch J, Karpelowsky JS, Darlington AE, Cohn RJ, Signorelli C, ANZCHOG Survivorship Study Group (2019). Paediatric surgery for childhood cancer: lasting experiences and needs of children and parents. Eur J Cancer Care (Engl).

[8] Hart JL, Turnbull AE, Oppenheim IM, Courtright KR (2020). Family-centered care during the COVID-19 Era. J Pain Symptom Manage.

[9] National Guideline Centre (2020). Evidence Review for Information and Support Needs: Perioperative Care in Adults: Evidence Review A.

[10] Leske JS (1996). Intraoperative progress reports decrease family members' anxiety. AORN Journal.

[11] Newton L, Sulman C (2018). Use of text messaging to improve patient experience and communication with pediatric tonsillectomy patients. Int J Pediatr Otorhinolaryngol.

[12] Jordan AL, Rojnica M, Siegler M, Angelos P, Langerman A (2014). Surgeon-family perioperative communication: surgeons' self-reported approaches to the "surgeon-family relationship". J Am Coll Surg.

[13] Insitute for Patient and Family Centered Care Patient- and Family-Centered Care.

[14] Lerman Y, Kara I, Porat N (2011). Nurse liaison: the bridge between the perioperative department and patient accompaniers. AORN J.

[15] Herd HA, Rieben MA (2014). Establishing the surgical nurse liaison role to improve patient and family member communication. AORN J.

[16] Stefan KA (2010). The nurse liaison in perioperative services: a family-centered approach. AORN J.

[17] Micheli AJ, Curran-Campbell S, Connor L (2010). The evolution of a surgical liaison program in a children's hospital. AORN J.

[18] Hanson-Heath CA, Muller LM, Cunningham MF (2016). Evaluating enhancements to a perioperative nurse liaison program. AORN J.

[19] Muldoon M, Cheng D, Vish N, Dejong S, Adams J (2011). Implementation of an informational card to reduce family members' anxiety. AORN J.

[20] Egeth M, Soosaar J, Shames A, Margolies R, Gurnaney H, Rehman M (2013). Operative heuristics: optimizing perioperative status boards. Biomed Instrum Technol.

[21] Andrews SM (2009). Patient family-centered care in the ambulatory surgery setting. J Perianesth Nurs.

[22] Koçyiğit M, Yilmaz G, Aksoy UM (2020). STAI test for evaluating the effect of showing surgery videos in the preoperative period on the anxiety levels of parents of children undergoing adenoidectomy or adenotonsillectomy. KBB ve BBC Dergisi.

[23] Carter AJ, Deselms J, Ruyle S, Morrissey-Lucas M, Kollar S, Cannon S, Schick L (2012). Postanesthesia care unit visitation decreases family member anxiety. J Perianesth Nurs.

[24] Statistics Canada Smartphone Personal Use and Selected Smartphone Habits by Gender and Age Group.

[25] Lesher AP, Gavrilova Y, Ruggiero KJ, Evans HL (2021). Surgery and the smartphone: can technology improve equitable access to surgical care?. J Surg Res.

[26] Lu K, Marino NE, Russell D, Singareddy A, Zhang D, Hardi A, Kaar S, Puri V (2018). Use of short message service and smartphone applications in the management of surgical patients: a systematic review. Telemed J E Health.

[27] Buck C, Keweloh C, Bouras A, Simoes EJ (2021). Efficacy of short message service text messaging interventions for postoperative pain management: systematic review. JMIR Mhealth Uhealth.

[28] Schwebel FJ, Larimer ME (2018). Using text message reminders in health care services: a narrative literature review. Internet Interv.

[29] Farias N, Rose-Davis B, Hong P, Wozney L (2020). An automated text messaging system (Tonsil-Text-To-Me) to improve tonsillectomy perioperative experience: exploratory qualitative usability and feasibility study. JMIR Perioper Med.

[30] Yang JY, Lee H, Zhang Y, Lee JU, Park JH, Yun EK (2016). The effects of tonsillectomy education using smartphone text message for mothers and children undergoing tonsillectomy: a randomized controlled trial. Telemed J E Health.

[31] Rodríguez-Huerta MD, Álvarez-Pol M, Fernández-Catalán ML, Fernández-Vadillo R, Martín-Rodríguez M, Quicios-Dorado B, Díez-Fernández A (2019). An informative nursing intervention for families of patients admitted to the intensive care unit regarding the satisfaction of their needs: The INFOUCI study. Intensive Crit Care Nurs.

[32] Gordon CR, Rezzadeh KS, Li A, Vardanian A, Zelken J, Shores JT, Sacks JM, Segovia AL, Jarrahy R (2015). Digital mobile technology facilitates HIPAA-sensitive perioperative messaging, improves physician-patient communication, and streamlines patient care. Patient Saf Surg.

[33] Kwan MK, Chiu CK, Gan CC, Chan CYW (2016). Can intraoperative text messages reduce parental anxiety of children undergoing posterior spinal fusion surgery for adolescent idiopathic scoliosis?. Spine (Phila Pa 1976).

[34] Poudel RR, Singh VA, Yasin NF (2020). The effect of intra-operative text messages in reducing anxiety levels among family members of patients undergoing major musculoskeletal tumour surgery. Indian J Orthop.

[35] Wieck MM, Blake B, Sellick C, Kenron D, DeVries D, Terry S, Krishnaswami S (2017). Utilizing technology to improve intraoperative family communication. Am J Surg.

[36] Howe LS, Wigmore D, Nelms N, Schottel P, Bartlett C, Halsey D, Krag M, Lunardini D, Monsey R, Beynnon B, Blankstein M (2021). Perioperative family updates reduce anxiety and improve satisfaction: a randomized controlled trial. J Patient Cent Res Rev.

[37] OR Manager Secure Apps Loop in Families during Surgery.

[38] Beauplat J (2021). La technologie transforme l'expérience du patient au CHUM. La Presse.

[39] Institute for Healthcare Improvement Science of Improvement: How to Improve.

[40] Associates in Process Improvement.

[41] Brunet F, Malas K (2019). L'innovation en santé: Réfléchir, agir et valoriser, 2e ed.

[42] Coleman KD, Chow Y, Jacobson A, Hainsworth KR, Drendel AL (2021). An evaluation of short anxiety measures for use in the emergency department. Am J Emerg Med.

[43] BinDhim N, Shaman A, Alhawassi T (2013). Confirming the one-item question Likert scale to measure anxiety. Internet J Epidemiol.

[44] HeathCare G Centricity™ Opera.

[45] Twilio Twilio Customer Engagement Platform: Intelligent Customer Engagement—at Scale.

[46] Dong Y, Peng CJ (2013). Principled missing data methods for researchers. Springerplus.

[47] Kang H (2013). The prevention and handling of the missing data. Korean J Anesthesiol.

[48] World Health Organization Global Strategy on Digital Health 2020-2025.

[49] Globus O, Leibovitch L, Maayan-Metzger A, Schushan-Eisen I, Morag I, Mazkereth R, Glasser S, Kaplan G, Strauss T (2016). The use of short message services (SMS) to provide medical updating to parents in the NICU. J Perinatol.

[50] Bournes DA, Mitchell GJ (2002). Waiting: the experience of persons in a critical care waiting room. Res Nurs Health.

[51] Joseph HK, Whitcomb J, Taylor W (2015). Effect of anxiety on individuals and caregivers after coronary artery bypass grafting surgery: a review of the literature. Dimens Crit Care Nurs.

[52] Singh Solorzano C, Steptoe A, Leigh E, Kidd T, Jahangiri M, Poole L (2019). Pre-surgical caregiver burden and anxiety are associated with post-surgery cortisol over the day in caregivers of coronary artery bypass graft surgery patients. Int J Behav Med.

[53] Robley L, Ballard N, Holtzman D, Cooper W (2010). The experience of stress for open heart surgery patients and their caregivers. West J Nurs Res.

[54] Norman DA The Psychology of Waiting Lines.

[55] Rodriguez JA, Clark CR, Bates DW (2020). Digital Health Equity as a Necessity in the 21st Century Cures Act Era. JAMA.

[56] Meyer VM, Benjamens S, Moumni ME, Lange JFM, Pol RA (2022). Global overview of response rates in patient and health care professional surveys in surgery: a systematic review. Ann Surg.

[57] Burgard T, Bošnjak M, Wedderhoff N (2020). Response rates in online surveys with affective disorder participants. Z fur Psychol.

[58] Sammut R, Griscti O, Norman I (2021). Strategies to improve response rates to web surveys: a literature review. Int J Nurs Stud.

[59] Martínez-Pérez B, de la Torre-Díez I, López-Coronado M (2015). Privacy and security in mobile health apps: a review and recommendations. J Med Syst.

[60] Dexter F, Epstein RH (2001). Reducing family members’ anxiety while waiting on the day of surgery: systematic review of studies and implications of HIPAA health information privacy rules. J Clin Anesth.

