# Correction: Automated Intraoperative Short Messaging Service Updates: Quality Improvement Initiative to Relieve Caregivers’ Worries

**DOI:** 10.2196/41052

**Published:** 2022-07-15

**Authors:** Alexandre Mignault, Éric Tchouaket Nguemeleu, Stephanie Robins, Éric Maillet, Edwige Matetsa, Stéphane Dupuis

**Affiliations:** 1 Bloc Opératoire Centre hospitalier de l’Université de Montréal Montréal, QC Canada; 2 Département des sciences infirmières Université du Québec en Outaouais St-Jérome, QC Canada; 3 École des sciences infirmières Faculté de médecine et des sciences de la santé Université de Sherbrooke Sherbrooke, QC Canada

In “Automated Intraoperative Short Messaging Service Updates: Quality Improvement Initiative to Relieve Caregivers’ Worries” (JMIR Perioper Med 2022;5(1):e36208) the authors noted one error.

In the originally published article’s *Acknowledgments* section, two names were inadvertently misspelled.

These names have now been corrected to “Carl Davidson-Desbiens” and “Mireille Dessureault” in the following statement:

Finally, we express our gratitude to Carl Davidson-Desbiens, Mireille Dessureault, and Sherley Durand from General Electric Healthcare as they adapted the operating room (OR) clinical information software and made this project possible.

The correction will appear in the online version of the paper on the JMIR Publications website on July 15, 2022, together with the publication of this correction notice. Because this was made after submission to PubMed, PubMed Central, and other full-text repositories, the corrected article has also been resubmitted to those repositories.

