# Supplementary material for: Automated Intraoperative Short Messaging Service Updates: Quality Improvement Initiative to Relieve Caregivers’ Worries
Source: JMIR Perioper Med. 2022 May 6;5(1):e36208. doi: 10.2196/36208 (PMC9084444; doi:10.2196/36208)
Supplement: Multimedia Appendix 1 [file periop_v5i1e36208_app1.pdf]

Vous avez reçu une série de messages textes récemment de la part du CHUM. Dans un souci permanent d'améliorer nos prestations et nos services, nous évaluons la qualité et la pertinence des informations qui vous ont été transmises.

Nous vous serions reconnaissants de bien vouloir compléter ce court sondage. Nous vous remercions d'avance de votre collaboration.

L'équipe du bloc opératoire

Partie A: Section générale

A1. Le nombre de messages reçus était adéquat.

- Totalement en accord ☐
- En accord ☐
- En désaccord ☐
- Totalement en désaccord ☐

A2. Combien de messages avez-vous reçu?

- 3 ☐
- 4 ☐
- 5 ☐
- 6 et plus ☐

A3. Les messages transmis étaient clairs.

- Totalement en accord ☐
- En accord ☐
- En désaccord ☐
- Totalement en désaccord ☐

A4. **Les messages transmis vous ont tenu(e) informé(e) sur l'avancement du parcours opératoire de votre proche.**

- Totalement en accord ☐
- En accord ☐
- En désaccord ☐
- Totalement en désaccord ☐

A5. **Les informations transmises dans les messages pendant la journée ont répondu à vos attentes et à vos besoins.**

- Totalement en accord ☐
- En accord ☐
- En désaccord ☐
- Totalement en désaccord ☐

A6. **Sur une échelle de 1 à 10, dans quelle mesure la réception de messages textes ont permis de réduire votre anxiété face au parcours opératoire de votre proche?**

(1 étant aucunement réduit et 10 étant grandement réduit)

- 1 ☐
- 2 ☐
- 3 ☐
- 4 ☐
- 5 ☐
- 6 ☐
- 7 ☐
- 8 ☐
- 9 ☐
- 10 ☐
- Ne s'applique pas ☐

Partie B: Section retour d'appel

B1. **Avez-vous noté que le numéro pour contacter la chirurgie d'un jour (514 890-8317) figurait dans le premier message?**

- Oui ☐
- Non ☐

B2. **Avez-vous eu besoin de contacter le service de chirurgie d'un jour suite à l'un des messages reçus?**

- Oui ☐
- Non ☐

B3. **Pour quelle(s) raison(s) avez-vous eu à contacter le service de chirurgie d'un jour?**

- Connaître un numéro de chambre ☐
- M'informer sur la durée du parcours opératoire ☐
- Complément d'information sur le parcours opératoire ☐
- Information sur l'état de santé de mon proche ☐
- Connaître son heure de congé de l'hôpital ☐
- Connaître l'adresse de l'hôpital ☐
- Autre ☐

Autre

Partie C: Section satisfaction générale

C1. **Sur une échelle de 1 à 5, combien évaluez-vous votre taux de satisfaction générale de l'application SMS?**

(1 étant complètement insatisfait, 5 étant complètement satisfait)

1 ☐
2 ☐
3 ☐
4 ☐
5 ☐

C2. **Suite à votre expérience avec le système de messagerie texte du bloc opératoire, avez-vous des suggestions et/ou commentaires?**

Merci de votre collaboration.

L'équipe de la chirurgie d'un jour, du bloc opératoire et de la salle de réveil du CHUM
