# Supplementary material for: Automated Intraoperative Short Messaging Service Updates: Quality Improvement Initiative to Relieve Caregivers’ Worries
Source: JMIR Perioper Med. 2022 May 6;5(1):e36208. doi: 10.2196/36208 (PMC9084444; doi:10.2196/36208)
Supplement: Multimedia Appendix 2 [file periop_v5i1e36208_app2.docx]

You recently received a series of text messages on behalf of the CHUM. In an ongoing effort to improve our services, we are evaluating the quality and relevance of the information you received.

We would be grateful if you would complete this short survey.

We thank you in advance for your cooperation.

The Operating Room Team

Section A: General information

A1. The number of SMS messages received was adequate

- completely agree
- agree
- disagree
- completely disagree

A2. How many messages did you receive?

- 3
- 4
- 5
- 6 or more

A3. The messages delivered were clear

- completely agree
- agree
- disagree
- completely disagree

A 4. The messages delivered kept me informed about the progress of my loved one’s surgical procedure

- completely agree
- agree
- disagree
- completely disagree

A 5. the information provided in the messages during the day met my needs and expectations.

- completely agree
- agree
- disagree
- completely disagree

A6.

On a scale of 1 to 10, to what level did receiving text messages reduce your anxiety about your loved one’s surgical journey? [with 1 being not at all reduced and 10 being greatly reduced].

- 1
- 2
- 3
- 4
- 5
- 6
- 7
- 8
- 9
- 10

Section B. Phone response

B1. Did you notice that the day surgery contact number (514-XXX-XXXX) was included in the

first message?

- yes
- no

B2. Did you need to contact the day surgery service despite having received SMS messages?

- yes
- no

B3. If yes what was the reason?

- To find out a room number,
- For information about the length of the operation
- For additional information about the operation
- For information about the condition of my loved one’s health
- For information concerning discharge time
- For the address of the hospital
- Other *(open field)*

Section C: General satisfaction

C1. On a scale of 1 to 5, how would you rate your overall satisfaction with the SMS application [with 1 being completely dissatisfied and 5 being completely satisfied].

- 1
- 2
- 3
- 4
- 5

C2. Do you have any suggestions and/or comments following their experience with the messaging system? *(open field)*

Thank you for your cooperation.

The CHUM Day Surgery, Operating Room and Recovery Room Team
